# Chronic gastrointestinal symptoms in adulthood revealing congenital intestinal malrotation with midgut volvulus: a case report

**DOI:** 10.1093/jscr/rjag693

**Published:** 2026-08-11

**Authors:** Mohammed A Maraqa, Danya Dawadi, Abdallah Badarna, Seema A Ghaith, Adam Al-Nahwi, Masa Zghyer, Rawan H Alhroub, Anwar Yousef Jabari

**Affiliations:** Surgical Department, Alia Governmental Hospital, Hebron, Palestine; Faculty of Medicine, Palestine Polytechnic University, Hebron, Palestine; Faculty of Medicine, Palestine Polytechnic University, Hebron, Palestine; Faculty of Medicine, Palestine Polytechnic University, Hebron, Palestine; Faculty of Medicine, Palestine Polytechnic University, Hebron, Palestine; Faculty of Medicine, Palestine Polytechnic University, Hebron, Palestine; Faculty of Medicine, Palestine Polytechnic University, Hebron, Palestine; Faculty of Medicine, Palestine Polytechnic University, Hebron, Palestine; Faculty of Medicine, Palestine Polytechnic University, Hebron, Palestine

**Keywords:** intestinal malrotation, volvulus, adulthood, Ladd procedure

## Abstract

Intestinal malrotation is a congenital anomaly of midgut rotation and fixation that typically manifests in infancy, with most cases diagnosed within the first year of life. Although rare, accounting for ~0.2%–0.5% of all malrotation cases, intestinal malrotation can present in adults, with nonspecific symptoms that may delay diagnosis. We report the case of a 48-year-old male with a four-year history of recurrent colicky abdominal pain and non-bilious vomiting that progressively worsened over the preceding three months, with vomiting increasing to nearly ten episodes daily. A contrast-enhanced abdominal computed tomography demonstrated marked dilatation of the proximal duodenum with a transition point at the mid-duodenum suggestive of a partial obstruction. Upper gastrointestinal fluoroscopy confirmed midgut malrotation with an incomplete volvulus. The patient subsequently underwent a Ladd procedure. Adult midgut malrotation should be considered in patients with chronic, unexplained abdominal pain and vomiting. Early diagnosis is essential to prevent life-threatening complications such as a volvulus and bowel ischemia.

## Introduction

Intestinal malrotation is defined as an abnormal rotation and fixation of the midgut during embryological development. This process typically takes place between the sixth and eighth weeks of gestation. Intestinal malrotation is estimated to occur in ~1 in 500 live births [1], with the majority of cases manifesting in infancy, which makes adult presentation quite uncommon. In adults, the prevalence of intestinal malrotation is estimated at 0.2%–0.5% of all reported cases [2].

Patients with malrotation often experience non-specific and chronic gastrointestinal complaints, including intermittent pain, vomiting, altered bowel habits, and weight loss. The clinical presentation is often subtle and atypical, frequently leading to a delayed diagnosis or misdiagnosis as more common gastrointestinal disorders. Patients with malrotation have a shortened mesenteric root, placing them at an increased risk for a midgut volvulus, which may result in significant morbidity and mortality [3].

We report the case of a 49-year-old man who has experienced gastrointestinal symptoms for a long time. This case highlights the diagnostic challenge of adult intestinal malrotation due to its rarity and nonspecific presentation, and emphasizes the importance of maintaining a high index of suspicion for congenital gastrointestinal anomalies in adults.

## Case presentation

A 48-year-old male patient presented to the emergency department with diffuse, colicky abdominal pain associated with recurrent vomiting of gastric contents. He had experienced multiple episodes over the previous four years, with a progressive worsening during the last three months. The vomiting has become more severe, occurring ~10 times daily. He also reported a history of altered bowel habits, including intermittent diarrhea. No alarming symptoms, such as fever, weight loss, hematemesis, or melena, were reported. The patient denied any prior abdominal surgeries or known chronic illnesses. His past medical and surgical history was unremarkable.

On physical examination, the abdomen was soft with mild distention, while vital signs were within normal limits. Routine laboratory investigations and abdominal radiography were performed upon admission.

**Figure 1:**
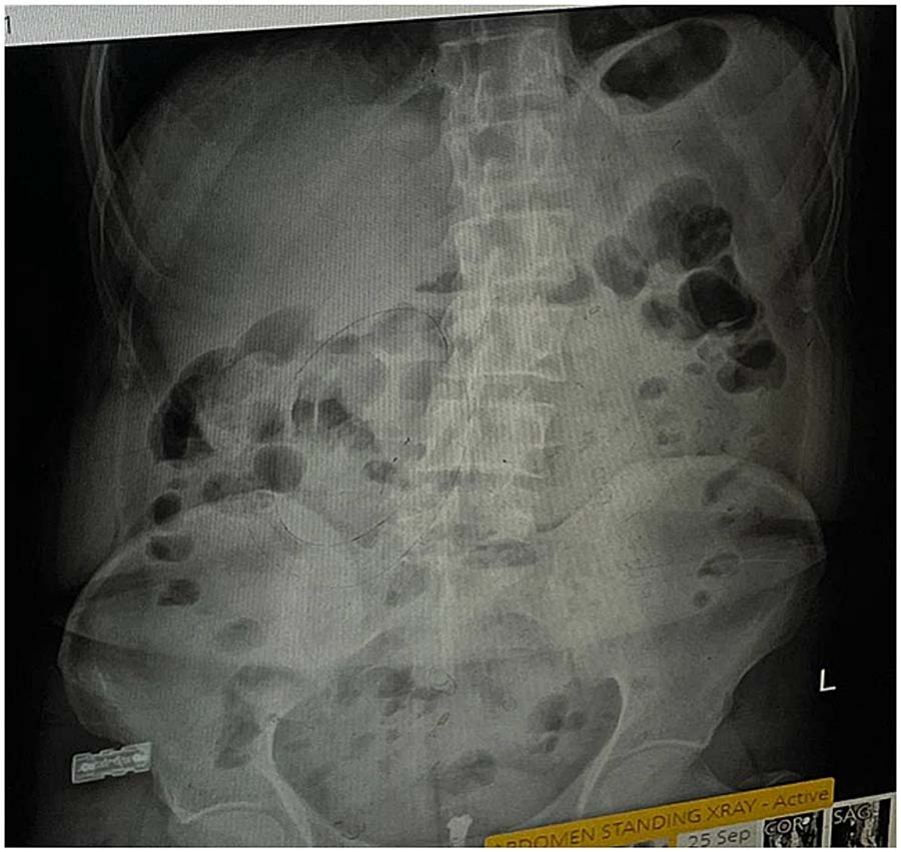
Erect abdominal X-ray demonstrating multiple dilated bowel loops with multiple air–fluid levels, consistent with intestinal obstruction.

An X-ray of the abdomen showed features of a bowel obstruction with multiple midgut air–fluid levels (Fig. 1). The patient was admitted and kept nil per os from midnight in preparation for a surgical intervention the following day. Prophylactic intravenous cefazolin (2 g) was administered before his transfer to the operating room.

The patient subsequently underwent a midline exploratory laparotomy under general anesthesia.

**Figure 2:**
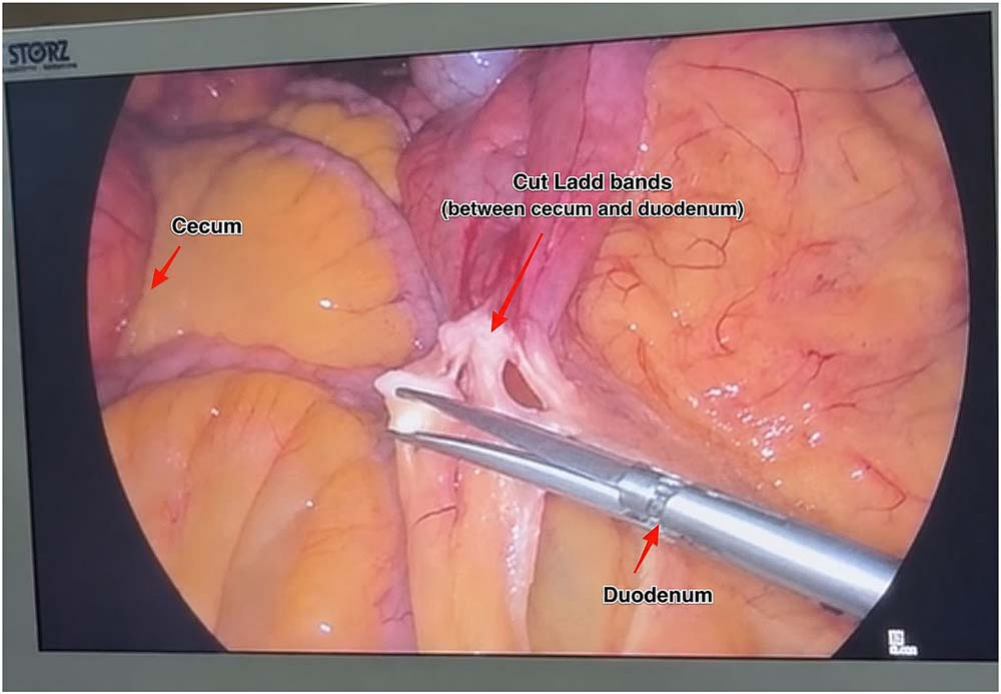
Laparoscopic view demonstrating Ladd bands extending between the cecum and duodenum, causing extrinsic compression. Division of the bands was performed.

Intraoperative findings revealed a duodenal obstruction secondary to intestinal malrotation associated with Ladd’s bands and a midgut volvulus. Division of the Ladd’s bands was performed to relieve the obstruction (Fig. 2). The midgut volvulus was carefully detorsed anticlockwise. To minimize the risk of a recurrent volvulus, the bowel was arranged in a non-rotation position, with the small intestine positioned on the right side of the abdomen and the colon on the left side.

**Figure 3:**
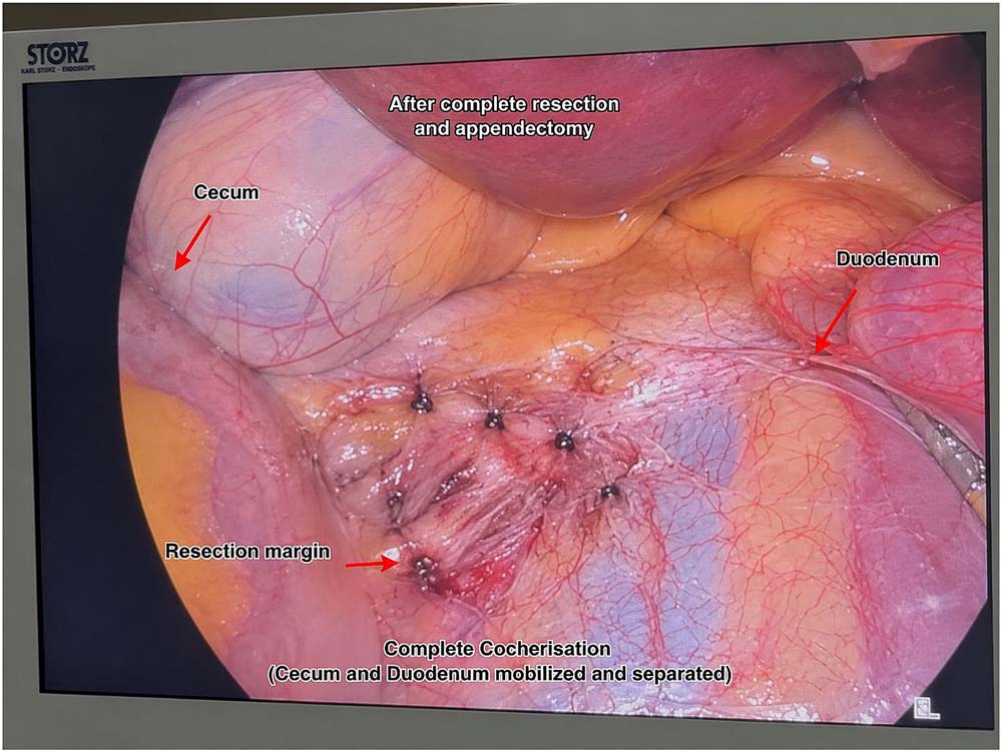
Post-procedural laparoscopic view after complete division of Ladd bands, appendectomy, and full Kocherization. Adequate mobilization and separation of the cecum and duodenum were achieved with satisfactory resection margins.

Given the abnormal anatomical location of the appendix, an appendectomy was performed to prevent potential future diagnostic confusion (Fig. 3). The abdominal cavity was irrigated, haemostasias was achieved, and the abdominal wall was closed in layers.

The postoperative course was notable for the development of subcutaneous emphysema and bilateral moderate pleural effusions, with adjacent lower-lobe passive atelectatic changes, on postoperative Day four. A right abdominal drainage tube was inserted, and mild postoperative pneumoperitoneum was also noted on imaging. The patient showed gradual clinical improvement and was discharged in a stable condition on postoperative Day five.

## Discussion

Intestinal malrotation is a congenital anomaly arising from incomplete rotation and fixation of the midgut between the 6th and 12th gestational weeks [4]. Although the estimated incidence at birth approaches 1 in 500 live births [4], more than 80%–90% of cases are diagnosed during infancy, with adult presentation representing only 0.2%–0.5% of reported cases [4, 5]. This epidemiologic discrepancy contributes to low diagnostic suspicion in adult surgical practice [5].

Adult malrotation demonstrates three principal clinical phenotypes described in contemporary literature [4]: incidental radiologic discovery [5]; chronic intermittent gastrointestinal symptoms [4, 5]; and acute obstruction or volvulus requiring emergency surgery [6, 7]. Published case series indicate that chronic symptoms may persist from months to several years prior to diagnosis [4, 6]. Our patient’s four-year history of recurrent colicky pain and vomiting aligns with the chronic-progressive phenotype, with an eventual transition to acute obstructive exacerbation, supporting the concept of intermittent midgut volvulus with spontaneous detorsion [6].

Radiologic assessment plays a decisive diagnostic role. Contrast-enhanced computed tomography (CT) has been reported to demonstrate a diagnostic accuracy between 80% and 97% in adult malrotation [5]. Characteristic findings include an abnormal right-sided small bowel, a left-sided colon, an abnormal position of the duodenojejunal junction, inversion of the superior mesenteric artery and vein, and the ‘whirlpool sign’ when a volvulus is present [5, 6]. In comparative adult reports, CT has largely replaced upper gastrointestinal contrast studies as the first-line modality due to superior vascular visualization and operative planning value [5]. In the present case, CT findings of proximal duodenal dilatation with a transition point correlated precisely with the intraoperative identification of Ladd’s bands and a volvulus, reinforcing the imaging–surgical concordance emphasized in recent literature [5, 7].

From a pathophysiological standpoint, the narrow mesenteric base inherent to malrotation predisposes to torsion [4]. Adult patients appear more likely to experience intermittent or partial volvulus compared with neonates, potentially explaining the prolonged symptom duration prior to catastrophic ischemia [6]. However, once a complete volvulus develops, morbidity mirrors pediatric cases, including the risk of bowel necrosis and short bowel syndrome if intervention is delayed [6, 8].

The Ladd procedure remains the definitive operative strategy across age groups [4, 6, 8]. Core components include counterclockwise detorsion, division of Ladd’s bands, widening of the mesenteric base, nonrotation repositioning (small bowel on the right, colon on the left), and a prophylactic appendectomy [4]. Adult cohort analyses report favorable symptom resolution following both open and laparoscopic approaches [6]. However, in acute obstructive or volvulus presentations, an open laparotomy is frequently preferred due to concern for compromised bowel viability and distorted anatomy [6, 7]. Our operative approach was therefore consistent with current adult surgical recommendations [6].

A mini-comparative synthesis of recent adult reports reveals several patterns: prolonged pre-diagnostic symptom intervals (often >1 year) [4, 6]; CT as the dominant diagnostic modality [5]; the Ladd procedure as universal definitive management [1, 6]; and low but incompletely defined recurrence rates in adults compared to pediatric populations (2%–7% reported in children) [6]. The present case contributes to this body of evidence by illustrating a prolonged symptomatic window with objective radiologic obstruction prior to acute deterioration—highlighting a potentially preventable phase of disease progression.

Notably, many published adult cases describe either incidental findings or sudden, catastrophic volvulus [5, 7]. Fewer reports detail the intermediate, chronic-obstructive phenotype with progressive symptom escalation and documented imaging correlation. This reinforces the educational value of the current case in emphasizing early cross-sectional imaging for adults with recurrent, unexplained vomiting and obstructive features.

Limitations include the inherent restriction of single-case evidence and limited long-term follow-up data. Nonetheless, the clear clinicoradiologic correlation and operative confirmation strengthen its contribution to the evolving understanding of adult malrotation patterns.

## Conclusion

Adult intestinal malrotation is a rare but clinically significant cause of chronic and acute intestinal obstruction. Recognition of the chronic-intermittent phenotype is essential to avoid a delayed diagnosis and prevent progression to a complete volvulus. Contrast-enhanced CT is the cornerstone of diagnosis and operative planning in adults. The Ladd procedure remains the gold-standard treatment, with favorable outcomes when performed prior to ischemic compromise. Increased awareness of the prolonged symptomatic window demonstrated in this case may contribute to earlier intervention and improved surgical outcomes.

## Data Availability

The data used to support the findings of this study are included in the article.
